# The Pro-Proliferative Effects of Nicotine and Its Underlying Mechanism on Rat Airway Smooth Muscle Cells

**DOI:** 10.1371/journal.pone.0093508

**Published:** 2014-04-01

**Authors:** Fang He, Bing Li, Zhuxiang Zhao, Yumin Zhou, Guoping Hu, Weifeng Zou, Wei Hong, Yimin Zou, Changbin Jiang, Dongxing Zhao, Pixin Ran

**Affiliations:** 1 Guangzhou Institute of Respiratory Diseases, The First Affiliated Hospital, Guangzhou Medical University, Guangzhou, Guangdong, China; 2 The Research Center of Experiment Medicine, Guangzhou Medical University, Guangzhou, Guangdong, China; William Harvey Research Institute, Barts and The London School of Medicine and Dentistry, Queen Mary University of London, United Kingdom

## Abstract

Recent studies have shown that nicotine, a major component of cigarette smoke, can stimulate the proliferation of non-neuronal cells. Cigarette smoking can promote a variety of pulmonary and cardiovascular diseases, such as chronic obstructive pulmonary disease (COPD), atherosclerosis, and cancer. A predominant feature of COPD is airway remodeling, which includes increased airway smooth muscle (ASM) mass. The mechanisms underlying ASM remodeling in COPD have not yet been fully elucidated. Here, we show that nicotine induces a profound and time-dependent increase in DNA synthesis in rat airway smooth muscle cells (RASMCs) in vitro. Nicotine also significantly increased the number of RASMCs, which was associated with the increased expression of Cyclin D1, phosphorylation of the retinoblastoma protein (RB) and was dependent on the activation of Akt. The activation of Akt by nicotine occurred within minutes and depended upon the nicotinic acetylcholine receptors (nAchRs). Activated Akt increased the phosphorylation of downstream substrates such as GSK3β. Our data suggest that the binding of nicotine to the nAchRs on RASMCs can regulate cellular proliferation by activating the Akt pathway.

## Introduction

Chronic Obstructive Pulmonary Disease (COPD) is currently the fourth leading cause of death worldwide and is expected to rise to the third leading cause. COPD is and will be a global health challenge in the next decades [1]. COPD is an inflammatory lung disease that is characterized by a progressive and largely irreversible airflow obstruction, which involves structural changes of the lung, including emphysema and small airway remodeling [2].

Cigarette smoking is one of the main risk factors for the development of COPD [3]. Cigarette smoke (CS) has previously been shown to induce features of airway remodeling in animal models, including airway wall thickening, increased ASM mass, goblet cell hyperplasia and collagen deposition [4], [5], [6], [7], [8]. Cigarette smoke contains many toxic constituents, including nicotine—the major addictive component in cigarette smoke that might play a more significant role than previously realized [9]. Recent studies have shown that nicotine can stimulate the proliferation of non-neuronal cells [10]. Nicotine stimulates proliferation in aortic smooth muscle cells, bronchial epithelial cells, and lung cancer cells via the nicotinic acetylcholine receptor [11], [12].

The activation of Akt by nicotine was detected in cultured normal airway cells and lung tumors [13]. Nicotine and its derivatives can regulate cellular proliferation and apoptosis by activating the Akt pathway [12], [13], [14]. The nicotine-induced proliferation of rat pulmonary artery smooth muscle cells is at least partially attributed to an up regulation of Cyclin D1 [15]. Akt, also known as protein kinase B (PKB), is a central node in a complex cascade of signaling pathways regulating cell proliferation, apoptosis, transcription and cell migration [16]. Akt triggers a network that positively regulates cell cycle progression through G1/S by inactivating GSK3β, which results in increased Cyclin D1.

Based on these observations, we hypothesized that nicotine might play an important role in the pathogenesis of COPD by activating the Akt pathway via the nAchRs on RASMCs. In this study, we demonstrate that nicotine could induce proliferative and early biochemical effects in RASMCs, such as the activation of the PI3K/Akt pathway. To assess the activation of Akt in an in vitro model system, we established primary RASMCs derived from large airways. The nicotine treatment activated Akt in the RASMCs at nanomolar doses within minutes. Multiple α and β subunits of the nAchRs, which bind to nicotine, were expressed in the RASMCs. Using pharmacologic inhibitors, we showed that the nicotinic activation of Akt depends upon PI3K and specific nAchRs. Once activated by nicotine, Akt increased the phosphorylation of downstream substrates, including GSK3β, and up regulated Cyclin D1 in vitro. Our results suggest that the subsequent steps resemble growth factor-induced cell proliferation, including the phosphorylation and inactivation of RB (retinoblastoma protein) and the enhanced recruitment of E2F to proliferative promoters. These events can be expected to contribute to the growth and progression of RASMCs exposed to nicotine through cigarette smoke.

## Materials and Methods

### Isolation of Rat Airway Smooth Muscle Cells

Male SD rats (4-8-weeks-old) were anesthetized with pentobarbital sodium (130 mg/kg i.p.). Entire isolated tracheas were rapidly removed and placed into cold HBSS. After dissecting the smooth muscle layer and removing the mucosal and connective tissues, the tracheal smooth muscle was chopped using a McIlwain tissue chopper at a setting of 500 μm. Primary rat airway smooth muscle cells were isolated by enzymatic digestion. The enzymatic digestion was performed using Ham's F12 medium containing 0.5% fetal bovine serum (FBS) supplemented with collagenase IV (2 mg/ml, Sigma Chemical, St. Louis, MO, USA), papain (1 mg/ml, Sigma Chemical, St. Louis, MO, USA). The cells were passaged by trypsinization using 0.25% trypsin, and only cells from passages 3–5 were used in subsequent experiments. The morphology of the isolated cells was assessed by immunofluorescence staining using a smooth muscle α-actin antibody. The purity of the cells was verified by laser-scanning confocal immunofluoresence microscope [14]. The cells were maintained in 25-cm^2^ flasks using Ham's F12 medium containing 10% fetal bovine serum (FBS), 100 U/ml penicillin and 100 mg/ml streptomycin, in a humidified atmosphere of 95% air and 5% CO_2_ at 37°C. The care and use of the animals was in compliance with regulations designated by the Chinese Association for Laboratory Animal Science Policy. All of the experimental protocols were approved by the Institutional Animal Care and Use Committee of Guangzhou Medical University.

### RASMCs Nicotine Exposure

For the dose-dependent induction of Akt phosphorylation, 100 μM, 10 μM, 1 μM, and 0.1 μM nicotine (Merck, Germany) was used to stimulate the RASMCs for 30 min. For the time-dependent induction of Akt phosphorylation, 10 μM nicotine was used. To assess the phosphorylation of downstream substrates, 10 μM nicotine was added for 30 min. For the in vitro kinase assays, 10 μM LY294002 (Sigma-Aldrich) or 1 μM SB216763 (Sigma-Aldrich) was added to the reactions 30 min prior to the addition of the 10 μM nicotine for 30 min. To assess the role of different nAchRs in mediating Akt activity, 10 μM nicotine was added for 30 min with or without a 30-min pretreatment with 100 μM MCA (Sigma-Aldrich) or 1 μΜ MG624 (Sigma-Aldrich). To examine of the effects of nicotine on cell proliferation, the RASMCs were pretreated for 30 min with 10 μM LY294002, 1 μM SB216763, 100 μM MCA, or 1 μM MG624, in addition to the treatment for 24 hours with 10 μM nicotine. The cells were harvested for cell counts, flow cytometry analysis, and EDU incorporation assays to evaluate the effects of nicotine on the cells undergoing DNA replication.

### Cell Counting

Cells were seeded in triplicate into 6-well plates at a density of 1×10^5^ cells per well and incubated overnight. The cells were then washed twice with PBS, and the media were changed to 0.5% FBS-containing medium for 24 h. The RASMCs were exposed to nicotine at various concentrations (100 μM, 10 μM, 1 μM, and 0.1 μM) for different treatment periods (0, 24, and 48 h). At the end-point, the cells were harvested by trypsinization and counted with a Zeiss Coulter Counter (Beckman Coulter, Miami, FL).

### Flow Cytometry Analysis

Cells were seeded in triplicate into 100-mm Petri dishes at a density of 1×10^5^ cells per plate and incubated overnight. The cells were then washed twice with PBS, and the media were changed to 0.5%-FBS-containing medium for 24 h. The RASMCs were exposed to nicotine at various concentrations (100 μM, 10 μM, 1 μM, and 0.1 μM) for different treatment periods (0, 24 h). The cells (1×10^6^) were harvested, washed twice with PBS, resuspended in 0.6 ml PBS, and fixed by the addition of 1.4 ml 100% ethanol at 4°C overnight. The fixed cells were rinsed twice with PBS, resuspended in propidium iodide (PI) solution containing 50 mg/ml PI and 50 mg/ml RNaseA (Sigma) in PBS, and incubated at 37°C for 30 min in the dark. The stained cells were analyzed using a FACScan Flow cytometer and Cell Quest analysis software (Becton Dickinson, San Jose, CA, USA). The flow cytometric analysis was repeated 3 times [17].

### RT-PCR

Total RNA was extracted from the RASMCs in each group using Trizol (Invitrogen). Efficient extraction was verified by electrophoresis on a 1.5% agarose gel and an absorbance (A260 / 280) value of 1.8–2.0. The complementary DNA was generated using the PrimeScript^TM^RT reagent KIT (Takara Biotechnology, Dalian, China). PCR was performed using an iCycler (Bio-Rad) with a touch-down program with annealing temperatures from 55°C to 53°C.Subunit-specific primers (Takara Biotechnology, Dalian, China) for the nAchRs were synthesized using the sequences below (Table 1).

**Table 1:** RT-PCR Primers.

Gene	Primer sequence (Sense/antisense)	Product size
α1 nAchR	5′-GACTACAGCAGTGTGGTCCG-3′ (sense)	490 bp
	5′-CCCACTCTCCGCTCTCCATG -3′ (antisense)	
α2 nAchR	5′- TCTGATGTGGTCATCGTGCG -3′ (sense)	610 bp
	5′-CAGTGAGGCAGGAGATGAGC-3′ (antisense)	
α3 nAchR	5′-TGAGGTGTCCATGTCTCAGC-3′ (sense)	725 bp
	5′-GGCATGGTGTGTGTGGTTGG-3′ (antisense)	
α4 nAchR	5′-CTGGGACCCTGGTGACTAC-3′ (sense)	380 bp
	5′-AGCACTCGTACTTCCTGGTG-3′ (antisense)	
α5 nAchR	5′-CTCTTCCTCCACACACAACGC-3′ (sense)	790 bp
	5′- CATGGTCCCAGCTACTCAGG-3′ (antisense)	
α6 nAchR	5′- CAACGGAGTACGATGGCATCG-3′ (sense)	250 bp
	5′- CAGCCTTGTCGTAAGTCCAGG-3′ (antisense)	
α7 nAchR	5′-GGTCCTGGTCCTATGGAGG-3′ (sense)	524 bp
	5′-GCAGAAACCATGCACACCAG-3′ (antisense)	
α9 nAchR	5′-CTCATCACCTGGGACTCACC-3′ (sense)	454 bp
	5′- GAGGCTGGCATGATCTCTGC-3′ (antisense)	
α10 nAchR	5′-CCTCACCTATGGCTGCTGCTC-3′ (sense)	514 bp
	5′- GCTTCCTGGTGGCATAGACAC-3′ (antisense)	
β1 nAchR	5′-AGGTCTGCCTCAGGAGCTAC-3′ (sense)	680 bp
	5′-GCTTACCAGACCTGCCATTCC-3′ (antisense)	
β2 nAchR	5′-ACTCACGGTGTTCCTGCTGC-3′ (sense)	550 bp
	5′-GGTCGATCACCATGGCAACG-3′ (antisense)	
β3 nAchR	5′-CCGTTTTGGTCTCTTTGACGG-3′ (sense)	582 bp
	5′-CAGAGAGTGGCTCCTAGTGG-3′ (antisense)	
β4 nAchR	5′-CACTGTCCCAGCTCATCAGTG-3′ (sense)	650 bp
	5′-GAGCAGCAGGAAGAACGTGAG-3′ (antisense)	

Unique primers for each nAchR subunit were used for the relevant PCR reactions. All of the results were sequenced and compared with the known subunit sequences to confirm that the correct subunit was being amplified.

### EDU Incorporation Assay

EDU incorporation assays were performed using the Cell-Light™ EDU imaging detecting kit, according to the manufacturer's protocols (RiboBio, Guangzhou, China). The RASMCs were seeded in triplicate into 96-well plates at a density of 1×104 cells per well and incubated in Ham's F12 medium containing 10% FBS overnight. The cells were then washed twice with PBS, and the media were changed to 0.1% FBS-containing medium for 24 h. The cells were next re-stimulated with 10 μM nicotine for 0, 12, 15, 18, 21, or 24 h. For the indicated experiments, 10 μM nicotine was added for 30 min with or without a 30-min pretreatment with 100 μM MCA, 1 μΜ MG624, 10 μM LY294002, or 1 μM SB216763. All of the EDU incorporation experiments were performed according to the kit's manufacturer's protocol [18], [19]. The EDU-positive cells were quantified by fluorescence microscopy. The assay was performed in triplicate and repeated three times. The proliferation rate of the RASMCs was calculated as the percentage of EDU-positive nuclei to total nuclei in five high-power fields per well [20].

### Immunocytochemistry and Immunofluorescence

The cells were seeded onto sterile round coverslips that had been placed inside 12-well plates. At 70% confluence, the medium was changed to 0.1% FBS-containing medium for 24 h. The cells were next re-stimulated with 10 μM nicotine for 0, or 12 h. All of the wells were washed twice with cold PBS, and a subset of the wells was fixed with 4% paraformaldehyde at room temperature for 20 min. The cells were then treated with 0.2% TritonX-100 (Sigma-Aldrich) at room temperature for 10 min. The cells on the coverslips were blocked in 2% donkey serum for 1 h at room temperature and then incubated with α-SMA mouse pAb (1∶200, Cell Signaling Technology, USA), Cyclin D1 mouse pAb (1∶100, Abcam, UK) over night at 4°C. The antibody binding was detected with a peroxidase-conjugated anti-mouse antibody according to the manufacturer's instructions; the slides were washed three times with cold PBS, and treated with the secondary antibody, donkey anti-mouse IgG (1∶250, Santa Cruz Biotechnology) for 1 h in the dark. The slides were again rinsed with PBS three times, mounted with 50% glycerol and stored in the dark. The immunofluorescence was examined using a Zeiss Axio Imager 2 Microscope (Carl Zeiss, Germany). The assay was performed in triplicate and repeated three times.

### Whole-Cell and Nuclear Protein Extraction and Western Blot

After the indicated pharmacological treatments described above, the whole-cell extracts were prepared by washing the treated cells with ice-cold PBS. The cells were lysed and incubated on ice in the presence of phosphatase and protease inhibitor cocktails (Keygenbio, Nanjing). To prepare nuclear extracts, first, the cells were collected in ice-cold PBS in the presence of Phosphatase Inhibitors to limit further protein modifications, then, the cells were resuspended in Hypotonic Buffer to swell the cell membrane and made it fragile. Addition of the Detergent caused leakage of the cytoplasmic proteins into the supernatant. After collection of the cytoplasmic fraction, the nuclei were lysed and the nuclear proteins were solubilized in the Lysis Buffer in the presence of the Protease Inhibitor Cocktail (Active Motif, USA). The protein concentrations were determined using a BCA Protein Assay Kit (Keygenbio, Nanjing). Forty micrograms of the protein samples was loaded and resolved on 10% SDS-PAGE gels and transferred to polyvinylidene fluoride membranes (Millipore, MA). The membranes were blocked in 5% nonfat milk/PBST (1× PBS, 0.10% Tween-20) for 1 h at room temperature and then incubated with primary antibodies (1× PBS, 5% nonfat milk, and 0.10% Tween-20; 1∶1,000 or 1∶500 antibody) overnight at 4°C. The membranes were subsequently washed with PBST three times and incubated with a peroxidase-conjugated secondary antibody diluted in 5% nonfat milk/PBST at room temperature for 1 h. The membranes were then washed in PBST. The immunoreactive bands were detected using an enhanced chemiluminescence system and Western blotting detection reagents (ECL) (Santa Cruz Biotechnology, USA). Densitometry of the immunoblotted bands was performed using the Quantity One image analysis software (Bio-Rad). The primary antibodies were Akt rabbit mAb (1∶1,000, Cell Signaling Technology, USA), phospho-Akt (Ser473)(p-Akt) rabbit mAb (1∶1,000, Cell Signaling Technology, USA),GSK3β rabbit mAb (1∶1,000, Cell Signaling Technology, USA), phospho-GSK3β (Ser9)(p-GSK3β) rabbit mAb (1∶1,000, Cell Signaling Technology, USA),Cyclin D1 rabbit mAb (1∶1,000, Cell Signaling Technology, USA),RB rabbit pAb (1∶500, Bioworld Technology, USA), phospho-RB (Ser807)(p-RB) rabbit pAb (1∶500, Bioworld Technology, USA). GAPDH mouse pAb (1∶1,000, JetWay Biotechnology, China), β-Tubulin mouse mAb (1∶500, Santa Cruz Biotechnology) and Lamin B mouse pAb (1∶2000, Biovision, USA) were used as loading standards. The secondary antibodies used were anti-rabbit IgG (1∶2,000; Santa Cruz Biotechnology) and anti-mouse IgG (1∶2,000, Santa Cruz Biotechnology). The immunoblot experiments were performed at least three times [21], [22].

### Statistical Analysis

All of the data are presented as the mean values ± the standard deviation (S.D.) from at least three independent experiments. The differences between two groups were analyzed by Student's t-test and between multiple groups by an ANOVA. The statistical analyses were performed using SPSS 13.0 software (Chicago, IL). *P*<0.05 was considered statistically significant.

## Results

### Nicotine Induces Proliferation-related Changes in RASMCs

**Figure 1:**
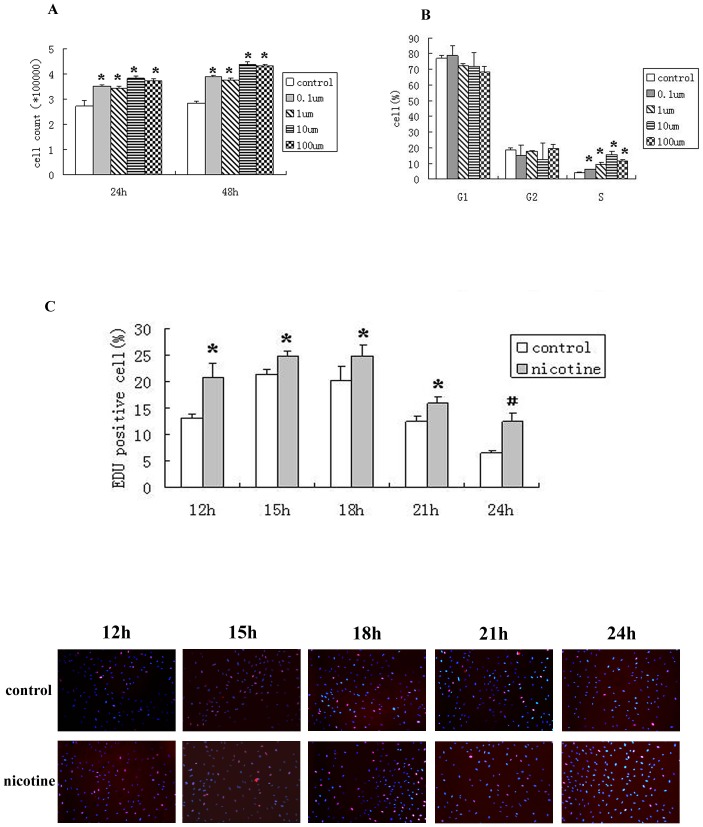
Nicotine promotes the proliferation of airway smooth muscle cells (RASMCs). (A) The cell numbers in the nicotine-treated groups were significantly increased, and the nicotine-mediated cell proliferation was time-dependent. (B) The percentage of the nicotine-treated cells in S-phase was significantly increased compared with the control group. (C) Nicotine promoted DNA replication; the DNA replication was increased by the nicotine treatment at every time point examined. The role of nicotine was particularly significant at the 24 h time point. *P<0.05, compared with the control group, N = 3; ^#^P<0.001, compared with the control group, N = 3.

To investigate the effects of nicotine on the proliferation of RASMCs in vitro, the RASMCs were stimulated with increasing concentrations of nicotine (0.1 μM–100 μM) for different treatment periods. The concentrations applied were equivalent to the doses experienced by smokers, which range from 0.2 μM to 100 μM [23], [24], [25]. We found that nicotine treatment significantly increased the RASMC cell numbers. There were statistically significant differences between the treated and control groups, and the nicotine-mediated cell proliferation was time-dependent (Figure 1A). Nicotine also affected the cell cycle—an increase in the percentage of cells in S-phase was maximally observed following treatment with 10 μM nicotine. Nicotine increased the percentage of S-phase cells significantly compared with the control group (Figure 1B). After the exposure to 10 μM nicotine for different treatment periods (0, 12, 15, 18, 21 or 24 h), an EDU incorporation assay was performed to detect the effects of the nicotine on the cells undergoing DNA replication. The nicotine increased the population of cells undergoing active DNA replication. Compared with the control group, active DNA replication was increased dramatically by nicotine at the 12- and 24-h time points (Figure 1C). These data suggest that nicotine induces proliferation-related changes in RASMCs.

### Nicotine Induces Cyclin D1 Activity and RB Phosphorylation

**Figure 2:**
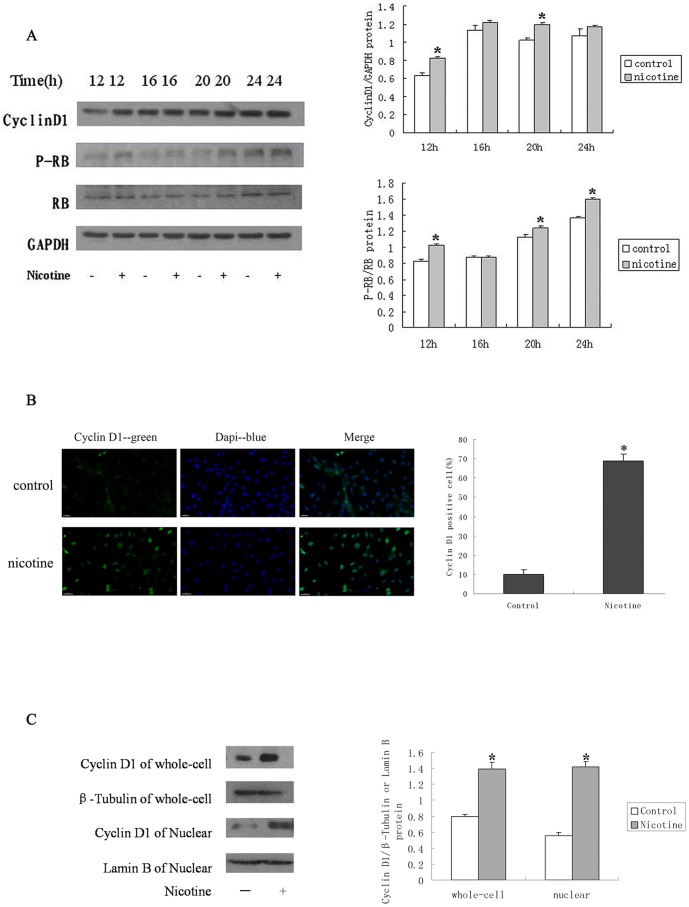
Nicotine Induces Cyclin D1 Activity and RB Phosphorylation. (A) Nicotine increased the levels of Cyclin D1 and phosphorylated RB, as indicated by Western blot. The expression of Cyclin D1 increased significantly in response to the nicotine treatment at the 12 and 20 h time points. Furthermore, nicotine induced the phosphorylation of RB and RB, which was maximal at 24 h. (B) The immunofluorescence analysis demonstrates the significant accumulation and nuclear localization of Cyclin D1 upon a 12-h nicotine treatment. (C) The western blot analysis shows that nicotine induces an significant increase in Cyclin D1 production at the 12 h time point examined in the whole-cell and nuclear extracts. β-tubulin was used as a loading control for the whole-cell fraction and Lamin B was used as a loading control for the nuclear fraction. *P<0.05, compared with the control group, N = 3; ^#^P<0.05, compared with the nicotine-treated group 10 μM, N = 3.

The activities of Cyclin D1 serve to integrate the extracellular signaling during the G1 phase with the cell cycle engine that regulates DNA replication and mitosis. The retinoblastoma protein (RB) plays a central role in regulating cell cycle progression and is inactivated in a wide variety of cancers [26], [27]. The inactivation of RB by kinases associated with Cyclin D1 and E facilitates the activation of E2F-regulated proliferative gene promoters, which promotes S-phase entry [27]. The effect of the nicotine stimulation on the Cyclin/CDK activity, as well as on RB function, was examined. To this end, we stimulated RASMCs with 10 μM nicotine for different treatment periods (0, 12, 16, 20, and 24 h). At the 12 h and 24 h time points, the exposure of the cells to nicotine significantly increased the Cyclin D1 and RB phosphorylation in a time-dependent manner by Western blot analysis (Figure 2A). In vitro kinase assays revealed that the kinase activity associated with Cyclin D1 was greatly enhanced upon nicotine stimulation, and the nicotine stimulation led to the accumulation and nuclear localization of Cyclin D1 (Figure 2B, 2C). Furthermore, the nicotine treatment resulted in the dissociation of E2F1 from RB that was correlated with the induction of RB phosphorylation. Thus, nicotine stimulation appeared to affect various components of the cell cycle machinery, similar to growth factor stimulation.

### Nicotine Induces the Proliferation of RASMCs via nAchRs

**Figure 3:**
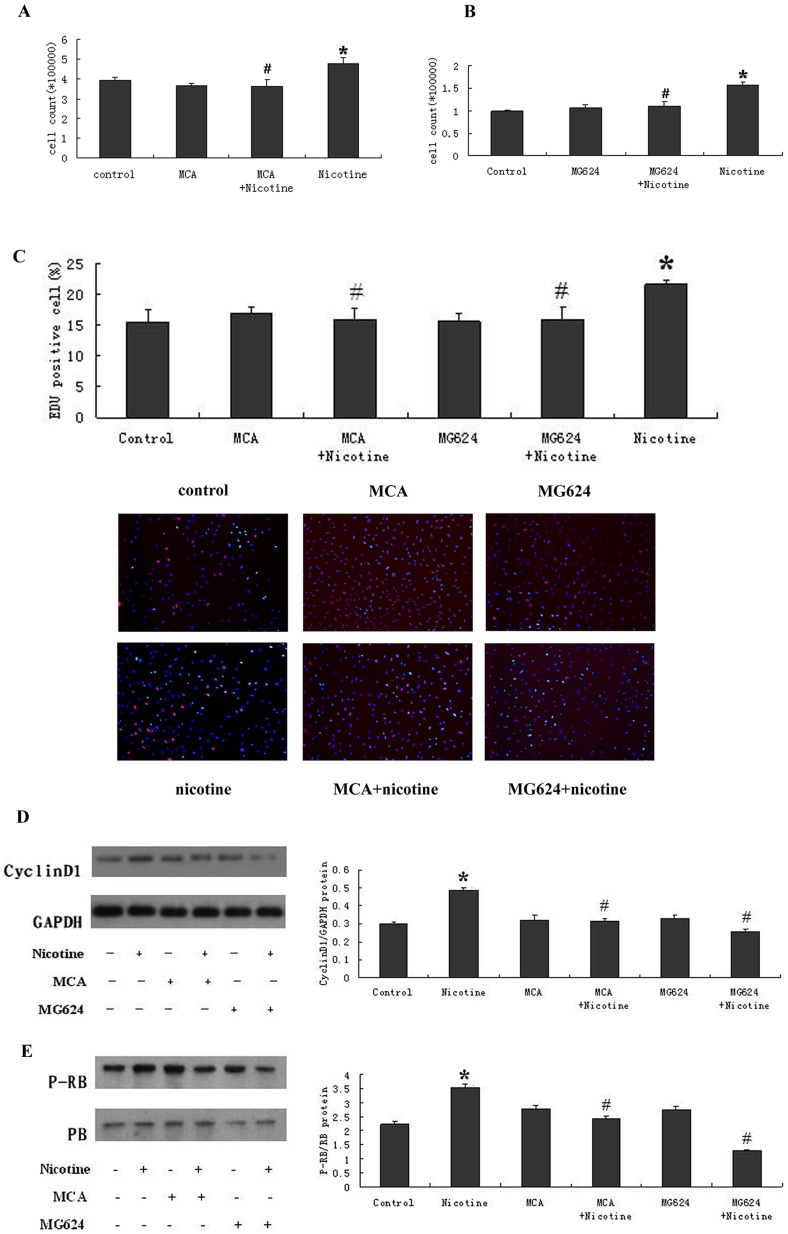
The effect of nAchRs on the nicotine-induced proliferation of RASMCs. (A, B) The RASMCs were treated with 10 μM nicotine for 24 h in the presence or absence of the nAchR subunit inhibitors MCA and MG624. The cells were harvested by trypsinization and then counted. The cell numbers in the nicotine- treated groups were significantly increased, whereas in the MCA + 10 μM nicotine-treated and MG624 +10 μM nicotine-treated groups, the cell numbers decreased significantly compared with the 10 μM nicotine-treated group. (C) Nicotine increased the levels of EDU incorporation at the 24 h time point. When the RASMCs were treated with nicotine in the presence of MCA or MG624, the percentage of S-phase cells was sharply reduced. (D) Nicotine increased the levels of Cyclin D1 at the 12 h time point, as detected by Western blot. When the RASMCs were treated with nicotine in the presence of MCA or MG624, the levels of Cyclin D1 decreased significantly compared with the nicotine-treated group. (E) Nicotine increased the level of RB phosphorylation at the 24 h time point as detected by Western blot. When the RASMCs were treated with nicotine in the presence of MCA or MG624, the levels of RB phosphorylation were significantly decreased compared with the nicotine-treated group. The role of MG624 was particularly significant. *P<0.05, compared with the control group, N = 3; ^#^P<0.05, compared with the nicotine-treated group (10 μM), N = 3.

The nAchRs belong to the superfamily of ligand-gated ion channels, which are predominantly expressed in neural tissues. However, the nAchRs have recently been reported to be expressed in other tissues [28], [29]. Upon nicotine binding, the nAchRs mediate the biologic effects of tobacco components. We examined the expression of the nAchR subunits in RASMCs. We performed nAchR subunit-specific RT-PCR analyses for the α1, α4, α7, α9, α10 and β1, β2, and β4 subunits in the RASMCs. To determine which nAchRs might facilitate the nicotine-induced proliferation of RASMCs, we treated the RASMCs with pharmacological inhibitors directed against the specific α subunit-containing nAchRs (the non-specific nAchR antagonist MCA and the α7 nAchR antagonist MG624) and measured proliferation-related changes in the RASMCs after nicotine treatment. Our results showed that the cell numbers were increased significantly in the 10 μM nicotine-treated group compared with the control-treated group. When the cells were treated in the presence of MCA or MG624, the cell numbers were decreased by 76% or 71%, respectively, compared with the 10 μM nicotine-treated group (Figure 3A, 3B). The EDU incorporation assays showed that the level of EDU incorporation in the control group was 15.42±2.23%, whereas a 10 μM nicotine treatment increased the population of S-phase cells to 21.55±0.83%. When the cells were treated in the presence of MCA or MG624, the percentages of S-phase cells decreased to 15.99±1.81% or 15.96±1.87%, respectively (Figure 3C). Similar results were obtained in our Western blot analysis. The exposure of the RASMCs to nicotine for 12 or 24 h resulted in a significant increase in Cyclin D1 expression and RB phosphorylation. In the presence of MCA or MG624, the expression of Cyclin D1 and the phosphorylation of RB were sharply decreased (Figure 3D, 3E). Our results indicated that the proliferative effects of nicotine require nAchR function. The nicotine-induced proliferation of the RASMCs was significantly inhibited by the non-specific nAchR antagonist MCA and the α7 nAchR antagonist MG624.

### Nicotine-induced The Activation of The Akt Pathway

**Figure 4:**
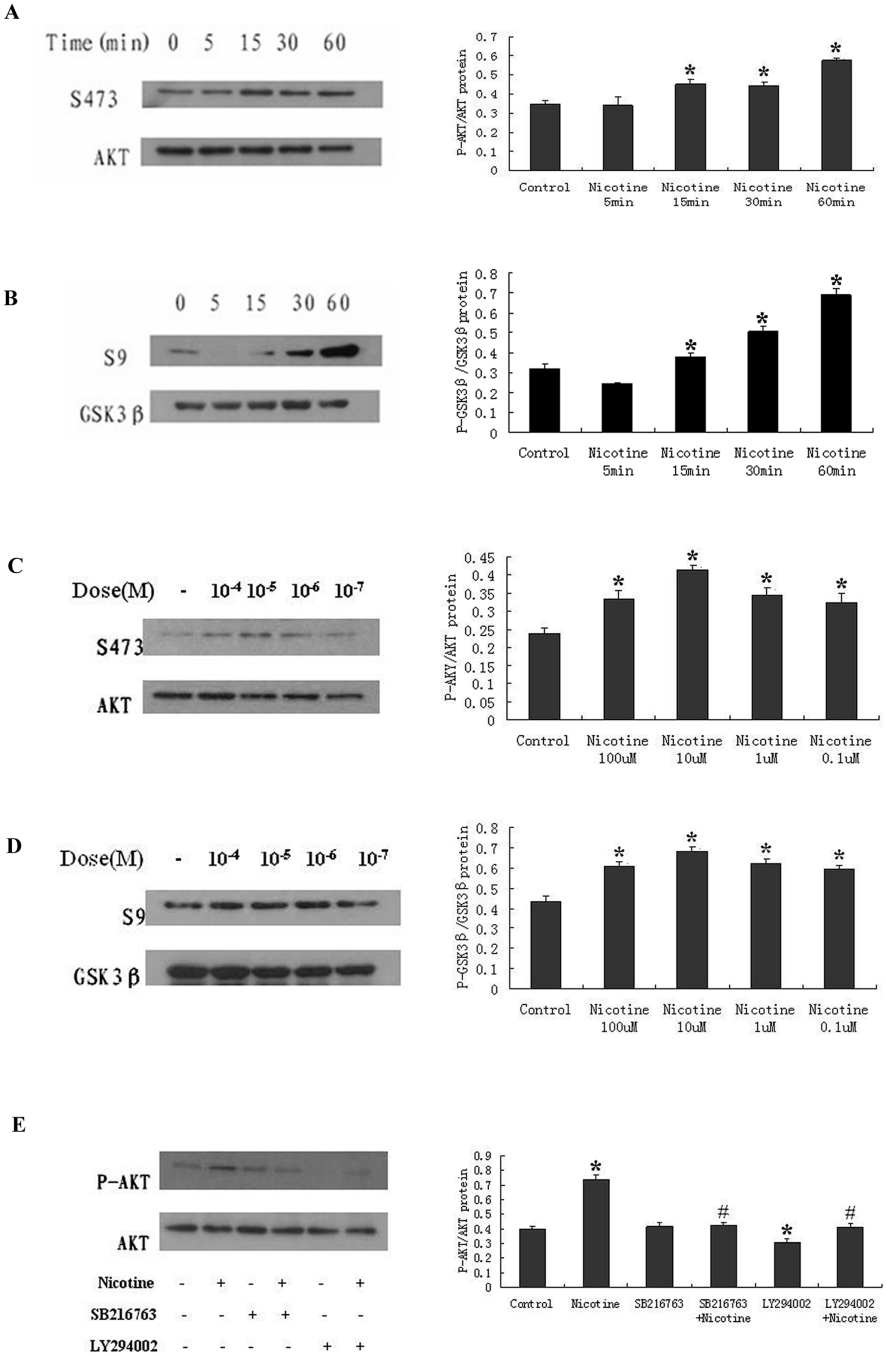
The nicotine-induced phosphorylation of Akt and GSK3β in RASMCs. (A, B) RASMCs were exposed to 10 μM nicotine for different treatment periods (0, 5, 15, 30, and 60 min). Nicotine treatment increased the Akt and GSK3β phosphorylation in a time-dependent manner in the RASMCs, as assessed by Western blot analysis. (C, D) RASMCs were exposed to the indicated concentrations of nicotine (0.1 μM–100 μM) for 30 min. The phosphorylation of Akt and GSK3β was increased by the nicotine treatment at every concentration examined. (E) RASMCs were treated with 10 μM nicotine for 30 min in the presence or absence of the PI3K inhibitor LY294002, or the GSK3β inhibitor SB216763, and the Akt phosphorylation was assessed by Western blot. Treatment with LY294002 +10 μM nicotine dramatically decreased the levels of phosphorylated Akt compared with treatment with 10 μM nicotine alone. In contrast, the GSK3β inhibitor SB216763 elicited no effect. When LY294002 was added to the culture medium without nicotine stimulation, the level of phosphorylated Akt was reduced compared with that of the control group. *P<0.05, compared with the control group, N = 3; ^#^P<0.05, compared with the nicotine group (10 μM), N = 3.

To investigate whether nicotine can activate Akt, we treated the RASMCs in vitro with various concentrations of nicotine (0.1 μM -100 μM) for different treatment periods (0, 5, 15, 30, or 60 min). Nicotine increased the phosphorylation of Akt at S473 (p-Akt) in a time-dependent manner (Figure 4A, 4C). The nicotine-stimulated phosphorylation of Akt in the RASMCs was evident within 15 minutes of the treatment and peaked at 60 minutes. In experiments designed to test the dose-dependent response to nicotine, we found that nicotine increased the phosphorylation of Akt at doses as low as 0.1 μM, and the maximum phosphorylation was observed at 10 μM (Figure 4C). To confirm that the increased Akt phosphorylation at S473 was indicative of increased kinase activity, we measured the phosphorylation of GSK3β, the downstream substrate of Akt. We found that the nicotine treatment increased the phosphorylation of GSK3β at S9 (p-GSK3β) in a time-dependent manner (Figure 4B, 4D), beginning as early as 30 min after the treatment. This dose-response relationship was similar to that of the nicotine-induced Akt phosphorylation in the RASMCs. Nicotine treatment increased the GSK3β phosphorylation at concentrations as low as 0.1 μM nicotine, but the maximum phosphorylation was observed with 10 μM nicotine (Figure 4D). The PI3K inhibitor LY294002 completely abrogated the nicotine-induced Akt phosphorylation (Figure 4E), indicating that PI3K mediates the nicotine-induced Akt phosphorylation in RASMCs. Our results indicated that nicotine induced the activation of Akt in a PI3K-dependent manner, which increased the phosphorylation of downstream substrates including GSK3β.

### Nicotine Induces The Activation of The Akt Pathway via nAchRs

**Figure 5:**
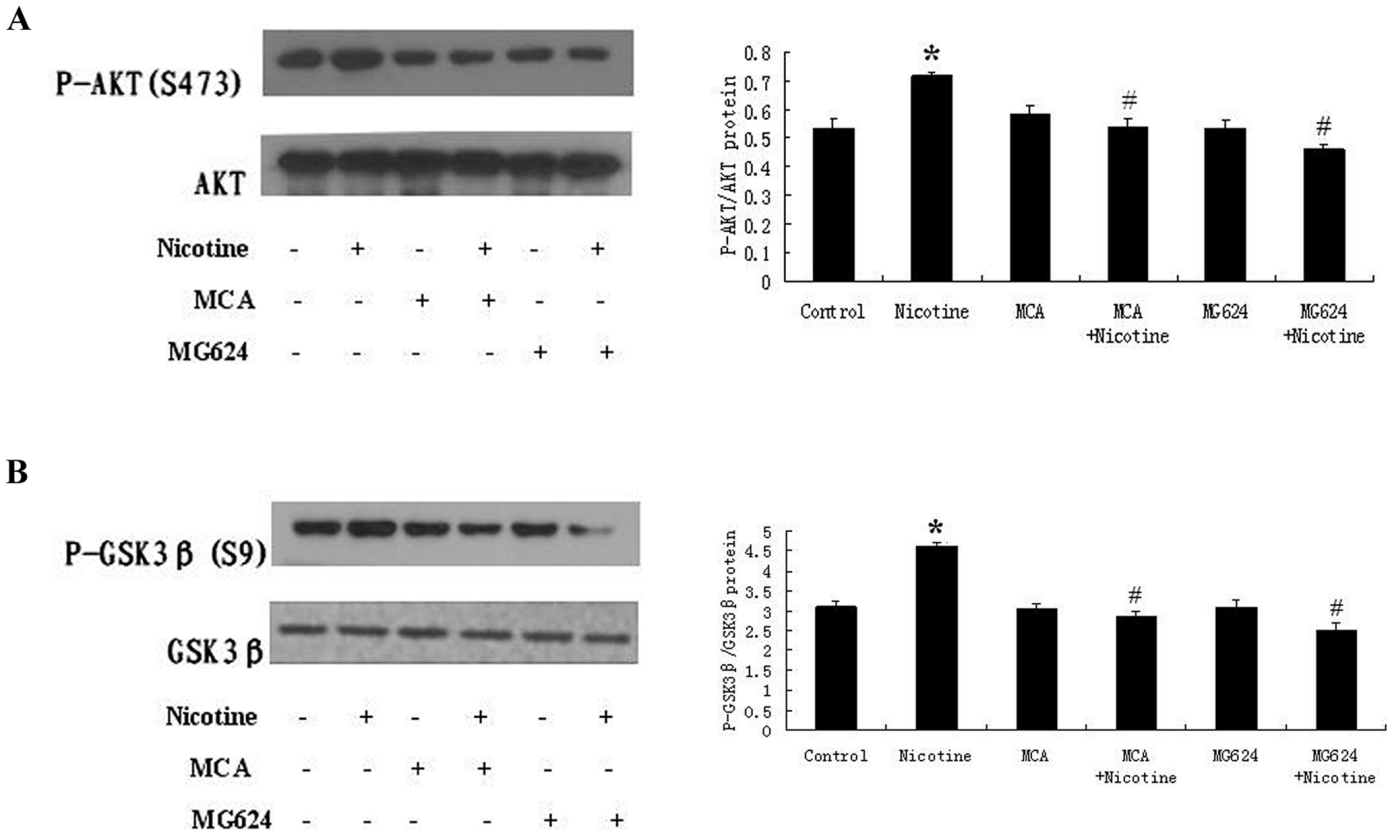
The effects of nicotinic antagonists on the activation of Akt in RASMCs. (A) RASMCs were treated with 10 μM nicotine for 30 min in the presence or absence of the nonspecific nAchR subunit inhibitor MCA and the α7-antagonist MG624. The levels of Akt phosphorylation in MCA +10 μM nicotine-treated and MG624 +10 μM nicotine-treated groups were decreased significantly compared with the nicotine-treated group. (B) RASMCs were treated with 10 μM nicotine for 30 min in the presence of MCA or MG624. The levels of GSK3β phosphorylation were also decreased; the role of MG624 was particularly significant. *P<0.05, compared with the control group, N = 3; ^#^P<0.05, compared with the nicotine-treated group (10 μM), N = 3.

To determine whether the nAchRs might mediate the nicotine-induced activation of Akt, we treated RASMCs with pharmacological inhibitors directed against specific α subunit-containing nAchRs (the nonspecific nAchR antagonist MCA and the α7 antagonist MG624) and measured the Akt activation after treatment with nicotine. Our results indicated that the levels of Akt and GSK3β phosphorylation in the MCA group and MG624 group were not significantly different when untreated with nicotine compared with the control group. However, the nAchRs antagonists MCA and MG624 inhibited the nicotine-induced phosphorylation of Akt and GSK3β. The levels of Akt and GSK3β phosphorylation of the MCA +10 μM nicotine-treated and the MG624 +10 μM nicotine-treated groups decreased significantly compared with the 10 μM nicotine-treated group. The inhibition of the α7 antagonist MG624 was particularly significant. These results suggest that nicotine activates the Akt pathway through the nAchRs (Figure 5A, 5B).

### Nicotine Promotes The Proliferation of RASMCs via Akt Pathway

**Figure 6:**
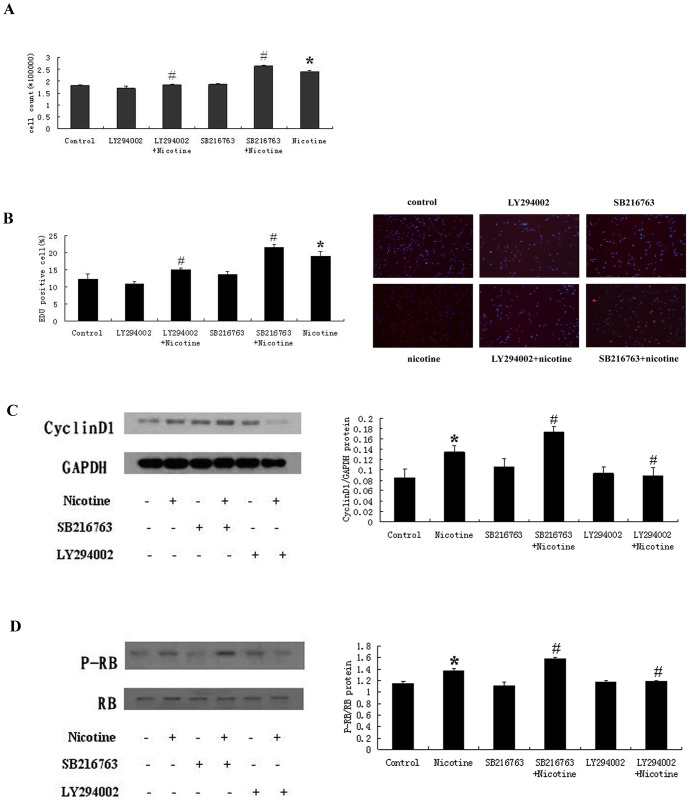
The effects of the Akt pathway on the nicotine-induced proliferation of RASMCs. (A) RASMCs were treated with 10 μM nicotine for 24 hours in the presence or absence of the PI3K inhibitor LY294002 or the GSK3β inhibitor SB216763. The cells were harvested by trypsinization and counted. In the LY294002 +10 μM nicotine-treated group, the cell numbers were significantly decreased, whereas the cell numbers in the SB216763 +10 μM nicotine-treated group increased significantly compared with the 10 μM nicotine-treated group. (B) RASMCs were treated with nicotine for 24 hours in the presence of LY294002. The EDU incorporation assays revealed that the percentage of S-phase cells dramatically decreased, whereas the percentage of S-phase cells in the SB216763 +10 μM nicotine-treated group increased compared with the nicotine-treated group. (C, D) RASMCs were treated with nicotine in the presence or absence of LY294002 or SB216763. The levels of Cyclin D1 expression at the 12 h time point and of RB phosphorylation at the 24 h time point were detected by Western blot analysis. The levels of Cyclin D1 expression and RB phosphorylation in the LY294002 +10 μM nicotine-treated group were significantly decreased, whereas they were significantly increased in the SB216763 +10 μM nicotine-treated group compared with the nicotine-treated group. *P<0.05, compared with the control group, N = 3; ^#^P<0.05, compared with the nicotine-treated group(10 μM), N = 3.

Activation of the PI3K/Akt pathway promotes cellular proliferation in multiple cell types [13], [30]. Recent studies have shown that nicotine can regulate cellular proliferation and apoptosis by activating the Akt pathway [12]. We tested whether the nicotine-induced activation of Akt is necessary for the proliferation of RASMCs. We treated RASMCs with the PI3K inhibitor LY294002 and the GSK3β inhibitor SB216763 and measured the proliferation-related changes in the RASMCs after treatment with nicotine. Our results showed that the treatment with 10 μM nicotine significantly increased the cell numbers compared with the control-treated group. When the cells were treated with nicotine in the presence of LY294002, the cell number was reduced from (2.42±0.04) ×10^5^ to (1.70±0.13) ×10^5^ compared with the 10 μM nicotine-treated group (Figure 6A). The EDU incorporation assays showed that the level of EDU incorporation in the control group was only 12.28±1.55% and increased to 19.05±1.33% upon 10 μM nicotine treatment. When the cells were treated with nicotine in the presence of LY294002, the percentage of S-phase cells was reduced to 14.94±0.58% (Figure 6B). Similar results were obtained in the Western blotting analysis. At 12 or 24 h after the treatment of the RASMCs with nicotine, the levels of Cyclin D1 expression and RB phosphorylation were significantly increased; when the cells were treated with nicotine in the presence of LY294002, the levels of Cyclin D1 expression and RB phosphorylation were dramatically reduced (Figure 6C, 6D). However, the effects of SB216763 were different from those of LY294002. When the cells were treated in the presence of SB216763, the cell numbers increased from (2.42±0.04) ×10^5^ to (2.64±0.04) ×10^5^ compared with the 10 μM nicotine treatment group (Figure 6A). The EDU incorporation assays showed that the SB216763 +10 μM nicotine treatments increased the percentage of S-phase cells from 19.05±1.33% to 21.60±0.83% compared with the 10 μM nicotine treatments (Figure 6B). Similar results were obtained in our Western blot analysis, which showed that the level of Cyclin D1 expression and RB phosphorylation was significantly increased by the SB216763 +10 μM nicotine treatment compared with the 10 μM nicotine treatment (Figure 6C, 6D). Our results showed that the nicotine-induced proliferation of the RASMCs was significantly inhibited by the PI3K inhibitor LY294002. The proliferative effects of nicotine required the activation of the Akt pathway.

## Discussion

COPD is a condition that is characterized by the airflow limitation in the peripheral airways that is not fully reversible and that generally becomes progressively worse over time [31]. A predominant feature of COPD is airway remodeling, which includes an increased airway smooth muscle (ASM) mass. The inhalation of noxious particles or gases, particularly cigarette smoke, represents a major risk factor.

Although the mechanisms involved in the development and progression of airway remodeling in COPD are largely unknown, chronic inflammation of the airways associated with the release of profibrotic cytokines and growth factors is presumably of major importance; the inflammatory changes are coupled to a repair or remodeling process that thickens the walls of these airways [32]. Recent studies have indicated that increased airway smooth muscle mass may contribute to the airway remodeling observed in COPD [2]–[5]. Airway remodeling could also result from the direct effects of cigarette smoke on ASMCs proliferation, independent of inflammation [33].

Cigarette smoke is a complex mixture of over 4,000 compounds; nicotine is one of the more important components of cigarette smoke, which was originally believed to only be responsible for tobacco addiction. It is also widely marketed as an aid to smoking cessation in the form of nicotine-replacement products. The nicotine supplements now used for smoking cessation include patches, nasal sprays, chewing gum, and the transdermal patches, these non-smoking nicotine delivery modalities can result in serum concentrations of nicotine that are equivalent to those observed in active smokers [34]. The impact and risks of long-term nicotine supplementation are unknown [35].

In human smokers, nicotine is not only found in blood plasma but also in saliva and induced sputum. Once inhaled, nicotine is quickly taken up by the blood stream and distributed throughout the body. The nicotine plasma concentration in active and passive smokers ranges between 1 μM and 0.01 μM. The nicotine concentrations found in saliva can be up to 10 μM during “smoking days” [36]. Five minutes after smoking a cigarette, the induced sputum contains a surprising 34 μM of nicotine [37]. Therefore, the lungs and bronchial surfaces of smokers might be exposed to significantly increased nicotine concentrations compared with that measured in the bloodstream [38]. Recent studies have shown that nicotine can stimulate the proliferation of non-neuronal cells. Nicotine stimulates proliferation in bronchial epithelial cells, lung cancer cells, and most notably in aortic smooth muscle cells [39], [40], [30], [22]. Further investigation into the biological activities of nicotine on ASMCs is warranted.

In this study, our in vitro data provide evidence that nicotine might contribute to airway remodeling through direct proliferative effects on RASMCs that might be involved in the increased ASM mass in COPD. We used nicotine concentrations of 100 μM-0.1 μM as representative concentrations for significant effects. The nicotine concentration that was demonstrated to induce significant proliferation of RASMCs was 10 μM. This conclusion is further supported by the discovery of up regulated Cyclin D1 expression and RB phosphorylation. In addition, the percentage of S-phase cells detected by flow cytometry and the level of replicating cells detected by EDU incorporation assay revealed an increase in S-phase cells, suggesting an enhanced G1/S phase transition of the RASMCs (Figure 1 and 2). Cyclin D1 is a critical regulator of cell cycle progression and plays a key role in controlling the G1/S transition [41]. Our study also demonstrates that nicotine treatment leads to the accumulation and nuclear localization of Cyclin D1; Cyclin D1 translocates into the nucleus, together with its binding partners CDK4 and CDK6, forms active complexes that promote cell cycle progression by phosphorylating and inactivating RB [42], [43], allowing E2F transcription factors to bind to proliferative gene promoters(Figure 2). The induction of the expression of these target genes facilitate S-phase entry and passage through the restriction point, leading to DNA synthesis [44]. Analysis of further downstream events also suggests that the mammalian cell cycle machinery is engaged in nicotine stimulation, analogous to growth factor stimulation.

Our study is the first to demonstrate that nicotine stimulates RASMCs proliferation through a functional nicotinic acetylcholine receptor. Once thought to be restricted to neuronal cells, the expression of the nAchR subunits has now been demonstrated in lung epithelial [13], [45], endothelial, aortic smooth muscle [39], SCLC [46], and NSCLC cells [30]. This wide distribution of nAchR expression might underlie the systemic physiological responses to smoking. There are several types of nAchR that are defined by the subunit composition of the receptor. The receptors can either contain only α-subunits, a combination of α and β subunits, or α, β, ε/γ, and δ subunits [47]. In our studies, we performed an nAchR subunit-specific RT-PCR analysis of the α1, α4, α7, α9, α10 and β1, β2, β4 subunits in RASMCs. The binding of nicotine to the nAchRs present on the surface of the RASMCs induces the proliferation of the RASMCs. The functional role of the nicotinic receptor in RASMCs has been confirmed by the inhibition of the nicotine-induced cell proliferation, resulting from the blockade of the nAchRs using specific antagonists (the non-specific nAchR antagonist MCA and the α7 antagonist MG624) (Figure 3). It is likely that more than one nAchR mediates the effects of nicotine, and this might explain the partial inhibitory effects of the antagonist. Our results are consistent with other reports on the different non-neuronal cell types. Our results, as well as the results from other groups, show that the α7 receptor subunit is essential for nicotine-mediated cell proliferation [39].

**Figure 7:**
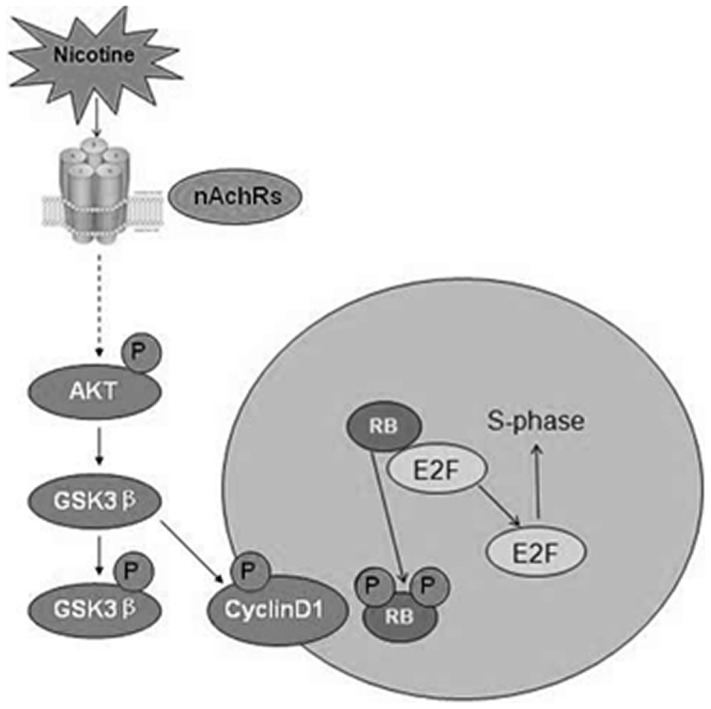
A schematic depicting the proliferative signaling induced by nicotine in RASMCs. Here, we show that nicotine functions similarly to a growth factor. The binding of nicotine to the nAchRs on RASMCs can regulate cellular proliferation by activating the Akt pathway. Akt triggers a network that positively regulates cell cycle progression through G1/S by phosphorylating and inactivating GSK3β, resulting in increased Cyclin D1. Cyclin D1 translocates into the nucleus and forms a holoenzyme with CDK4/6 to phosphorylate RB protein, resulting in the release of E2F. The E2F remains bound to the proliferative gene promoters, thereby facilitating S-phase entry. Thus, the signaling pathways induced by nicotine in the proliferation of RASMCs resemble those involved in growth factor stimulation. (This figure is referred to http://pathwaymaps.com/maps/474_map.png)

Nicotine is now recognized for its modulation of key cellular proteins, such as Akt, Cyclin D1, and other key cellular processes, such as proliferation and survival [48]. We demonstrated for the first time that the nicotine-induced proliferation of RASMCs is dependent on the phosphorylation of Akt and downstream mitogenic signaling (Figure 4 and 6). Nicotine has been reported to cause Akt (PKB) activation in cultured normal airway cells [30]. The activation of Akt by nicotine was detected in airway epithelial cell and lung tumors [13].The present study demonstrates that nicotine stimulates RASMC proliferation in an Akt-dependent manner. Nicotine induces the phosphorylation of Akt and GSK3β, as well as the increased expression of Cyclin D1 in RASMCs, whereas the inhibition of Akt can inhibit the nicotine-induced proliferation of RASMCs. The activation of Akt by nicotine phosphorylates GSK3β preventing the phosphorylation of Cyclin D1, which leads to the accumulation and nuclear localization of Cyclin D1, the activation of CDK4/6 and cell cycle progression(Figure 7). Akt triggers a network that positively regulates G1/S cell cycle progression by inactivating GSK3β, resulting in increased Cyclin D1 levels. The activity of GSK3β is inhibited by Akt-dependent phosphorylation. Thus, Akt-mediates the inhibition of GSK3β and stabilizes Cyclin D1 levels [15]. Cyclin D1 is a short-lived protein, the levels of which are regulated by phosphorylation-dependent proteolytic degradation that is regulated by the PI3K/Akt pathway. The activation of the PI3K/Akt pathway is required for progression through G1/S, and PI3K inhibition can induce a G1 arrest in many cell types [16]. Our results show that the nicotine-induced proliferation of RASMCs is significantly inhibited by the PI3K inhibitor LY294002. The concentrations used here were consistent with those reported by others [13]. Treatment with LY294002 significantly decreased the expression of Cyclin D1 and the phosphorylation of RB that were up regulated by nicotine (Figure 6). The fact that the pharmacological inhibition of Akt inhibited the nicotine-induced proliferation of RASMCs suggests that other signaling pathways that might be increased by nicotine stimulation do not significantly contribute to the induced proliferation in these cells, unless they are directly downstream of Akt.

The identification of the nAchR subunits in the RASMC lines provides a mechanistic basis for the activation of Akt in response to tobacco components (Figure 5). There is evidence supporting the nAchR-mediated nicotine involvement in intracellular processes that regulate the Ca^2+^ concentration, proliferation, and tubule formation of endothelial cells [49]. The stimulation of the nAchRs with nicotine enhances Ca^2+^ influx into the neurons. Of the several types of neuronal nAchRs, the α7 subunit-containing nAchRs exhibit high calcium permeability. PI3K and its downstream target Akt have been reported to be important for calcium-mediated survival in a wide variety of cells [50]. It will be interesting to study the involvement of Ca^2+^ in the context of nicotine-induced proliferation of RASMCs. The flux of Ca^2+^ through the nAchRs might represent the intrinsic link between nicotine, the nAchRs and the Akt pathway. Clearly, further studies are needed to clarify the role of nicotine in the proliferation of SMCs in airway remodeling. For example, the calcium-activated signaling and in vivo models might yield important information in this respect.

Taken together, our observations demonstrate that nicotine binding to the nAchRs on RASMCs can regulate cellular proliferation by activating the Akt pathway (Figure 7). This study elucidates a novel aspect of the pathogenesis of COPD and might open new avenues for the therapeutic targeting in COPD.
